# Microvesicle Profiles in Patients with HIV, HBV, and HCV Infections: An Exploratory Pilot Study

**DOI:** 10.3390/microorganisms14010124

**Published:** 2026-01-07

**Authors:** Georgios Dryllis, Sotirios P. Fortis, Nikolaos Martsoukos, Vasiliki Pantazatou, Evgenia Spyropoulou, Despoina Pontikaki, Christelos Kapatais, Nikolaos Tsakalis, Andrianna Konstantelou, Eleni Myrto Trifylli, Andreas G. Tsantes, Effie G. Papageorgiou, Serena Valsami, Andreas Kapatais, Olga Kosmopoulou, Anastasios G. Kriebardis

**Affiliations:** 1 Laboratory of Reliability and Quality Control in Laboratory Hematology (HemQcR), Department of Biomedical Sciences, School of Health & Caring Sciences, University of West Attica (UniWA), Ag. Spyridonos Street, 12243 Athens, Greecepantazatouvasiliki@gmail.com (V.P.); bisc21678301@uniwa.gr (E.S.);; 2 General Hospital of West Attica “Agia Varvara’’, 12351 Athens, Greecechristeloskapatais@hotmail.com (C.K.);; 3 1st Department of Internal Medicine, General Hospital of Nikea “Agios Panteleimon”, 18454 Piraeus, Greeceolgakosm@otenet.gr (O.K.); 4 Gastrointestinal-Liver Unit, The 2nd Department of Internal Medicine, National and Kapodistrian University of Athens, General Hospital of Athens “Hippocratio”, 11521 Athens, Greece; trif.lena@gmail.com; 5 Laboratory of Haematology and Blood Bank Unit, Attikon University Hospital, School of Medicine, National and Kapodistrian University of Athens, 12462 Athens, Greece; andreas.tsantes@yahoo.com; 6 Hematology Laboratory and Blood Bank, Aretaieion Hospital, National and Kapodistrian University of Athens, 11528 Athens, Greece

**Keywords:** microvesicles, chronic viral infections, human immunodeficiency virus (HIV), hepatitis B virus (HBV), hepatitis C virus (HCV)

## Abstract

Microvesicles (MVs) are extracellular vesicles released from many cell types under physiological and pathological conditions, influencing viral transmission, immune regulation, and inflammation. This exploratory pilot study characterized and compared plasma MV profiles in patients infected with human immunodeficiency virus (HIV), hepatitis B virus (HBV), and hepatitis C virus (HCV). Plasma samples (n = 125; HIV: 25, HBV: 50, HCV: 50) were analyzed using nanoparticle tracking analysis (NanoSight NS300) to assess MV size and concentration, classifying them as small (<300 nm) or large (>300 nm). Patients with HBV exhibited significantly larger mean MV size compared with both patients with HIV (131.5 ± 14.6 nm vs. 113.1 ± 14.0 nm, *p* < 0.0001) and HCV (131.5 ± 14.6 nm vs. 118.0 ± 18.5 nm, *p* = 0.0002). HCV infection showed higher concentrations of large MVs than HIV (*p* = 0.0022), while total and small MV levels did not differ. No sex-related differences were detected. Distinct MV size distributions appear linked to chronic viral infections, with HBV and HCV showing greater alterations than HIV. MVs may serve as potential biomarkers reflecting infection-associated biological processes; however, mechanistic, or functional roles were not assessed in this study and will require dedicated future investigations in larger controlled studies.

## 1. Introduction

Extracellular vesicles (EVs) are a diverse group of membrane-bound spherical particles that are shed by nearly all types of cells [1]. They are important mediators of intercellular communication via protein, lipid, and nucleic acid transfer between the parent and recipient cells [2]. Exosomes, microvesicles (MVs), and apoptotic bodies are the major subtypes of EVs with distinct sizes, biogenesis, and biological functions [1]. MVs are sized between 1 and 0.1 μm, arising from the shedding of the plasma membrane following cell activation, aging, or apoptosis [3,4].

A growing body of data suggests that EVs are intimately involved in a variety of biological processes, including regulation of immunity, coagulation, and inflammation, as well as in disease pathogenesis and progression [5,6]. In red blood cells (RBCs), MVs are required for reticulocyte maturation into erythrocytes, membrane damage repair, and clearance of invasive/damaging factors or oxidant ones that could otherwise induce alteration in structure and function [4,7,8]. In addition, MVs are implicated in intercellular communication and have also been involved in pathological states, including diabetes, chronic renal failure, and some cancers [9,10,11].

MVs are generated by different types of cells such as erythrocytes, leukocytes, platelets, and endothelial cells [4,8]. Their distribution in the circulation changes in periods of stress (either acute or chronic) and can exert both pro-oxidant and anti-oxidant effects, depending on the cellular environment [8,12]. Oxidative stress, in particular, has also been shown to modify MV production and composition, with altered MV profiles associated with different pathological conditions [4,12]. Furthermore, MVs are implicated in pro-thrombotic reactions, as they can activate platelets, macrophages, and neutrophils, while their overproduction has been reported in various types of cancer [5,6,10].

Due to their participation in a wide range of physiological and pathophysiological processes, reliable isolation and characterization protocols are necessary for further understanding of MV biology as well as for potential clinical applications. With the development of analysis tools, including mass spectrometry and nanoparticle tracking analysis (NTA), isolation and characterization have been refined to provide a more consistent population of microvesicles [13,14]. It is possible to measure the size distribution and number concentration of MVs in biological fluids, characterizing their Brownian motion with light scattering and video microscopy via NTA [15,16]. These technological advances open up new horizons for the exploration of MVs as potential biomarkers for disease diagnosis and prognosis [5,17].

Alterations in plasma MVs can also be found in viral infections such as hepatitis B (HBV), hepatitis C (HCV), and human immunodeficiency virus (HIV) [18,19]. More particularly, HCV is associated with elevated platelet-derived MVs and poor clinical outcomes [18], and even effective antiretroviral therapy does not decrease elevated levels of MV in HIV patients [19].

This study aimed to detect and quantify microvesicles in patients with hepatitis B, hepatitis C, and HIV, and to compare MV profiles among these groups in order to identify potential differences related to each viral infection.

## 2. Results

### 2.1. Descriptive Characteristics of the Research Sample

The research sample consisted of 40% patients with HBV infection (n = 50), 40% with HCV (n = 50) infection, and 20% with HIV infection (n = 25). Regarding gender distribution, 68.8% of participants were men and 31.2% were women. The average age of patients was 56.49 years, ranging from 32 to 84 years. The mean age of male patients was 56.69 years, while that of female patients was 56.05 years, with no statistically significant difference between genders (*p* = 0.799).

### 2.2. Distribution of Microvesicle Characteristics by Infection Type

**Figure 1:**
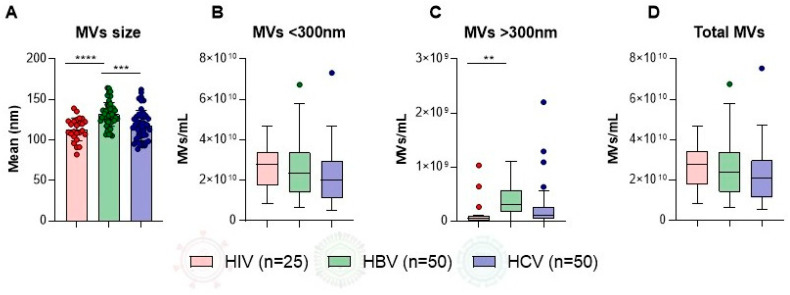
Comparison of MV characteristics among all (males and female) patients with HIV, HBV, and HCV infections. Bar and box plots illustrate the distribution of (**A**) MV size (nm) and concentrations (MVs/mL) for particles (**B**) smaller than 300 nm, (**C**) larger than 300 nm, and (**D**) total MVs. Error bars represent standard deviation, and asterisks indicate levels of statistical significance. (**** *p* < 0.0001; *** *p* < 0.001; ** *p* < 0.01). MV, microvesicle; HIV, human immunodeficiency virus; HBV, hepatitis B virus; HCV, hepatitis C virus; nm, nanometer; mL, milliliter; *p*, probability value.

The comparison of MV characteristics among patients with HIV, HBV, and HCV infections revealed significant differences in MV size and concentration. The mean MV size was significantly higher in patients with HBV compared to HIV (131.50 ± 14.63 nm vs. 113.10 ± 14.03 nm, *p* < 0.0001) and compared to HCV (131.50 ± 14.63 nm vs. 118.0 ± 18.47 nm, *p* = 0.0002) (Figure 1A). For microvesicles smaller than 300 nm, no statistically significant differences were found among the three infection groups (Figure 1B). However, for microvesicles larger than 300 nm, patients with HBV infection showed significantly higher concentrations compared to those with HIV infection (3.84 × 10^8^/mL vs. 1.16 × 10^8^/mL, *p* = 0.0022) (Figure 1C). The total MV concentration did not differ significantly between the groups (HIV: 2.68 × 10^10^/mL ± 1.08 × 10^10^/mL, HBV: 2.55 × 10^10^/mL ± 1.36 × 10^10^/mL, HCV: 2.14 × 10^10^/mL ± 1.28 × 10^10^/mL) (Figure 1D).

### 2.3. Distribution of Microvesicle Characteristics Among Male Patients by Infection Type

**Figure 2:**
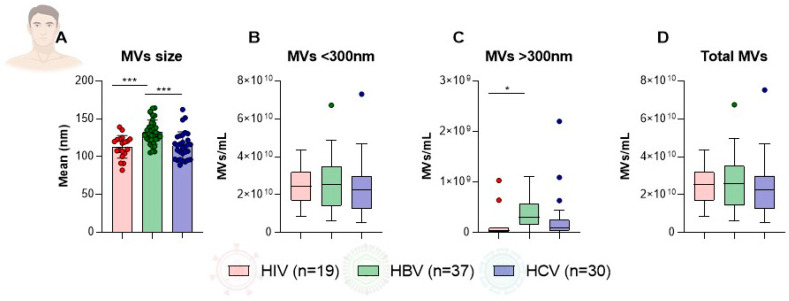
Comparison of plasma MV characteristics among male patients with HIV, HBV, and HCV infections. Bar and box plots illustrate the distribution of (**A**) MV size (nm) and concentrations (MVs/mL) for particles (**B**) smaller than 300 nm, (**C**) larger than 300 nm, and (**D**) total MVs. Error bars represent standard deviation, and asterisks indicate levels of statistical significance. (*** *p* < 0.001; * *p* < 0.05). MV, microvesicle; HIV, human immunodeficiency virus; HBV, hepatitis B virus; HCV, hepatitis C virus; nm, nanometer; mL, milliliter; *p*, probability value.

Among male patients with HIV, HBV, and HCV infections, significant differences were observed in microvesicle (MV) size and concentration. The mean size of MVs was significantly larger in men with HBV compared to those with HIV (132.30 ± 15.88 nm vs. 113.00 ± 14.96 nm, *p* = 0.0002) and HCV infection (132.30 ± 15.88 nm vs. 114.60 ± 18.19 nm, *p* = 0.0001), while no significant difference was found between the HIV and HCV groups (Figure 2A). Regarding MV concentrations, no significant differences were detected among the three groups for MVs smaller than 300 nm (HIV: 2.47 × 10^10^/mL ± 1.10 × 10^10^/mL, HBV: 2.63 × 10^10^/mL ± 1.31 × 10^10^/mL, HCV: 2.29 × 10^10^/mL ± 1.43 × 10^10^/mL) or for total MV concentration (HIV: 2.49 × 10^10^/mL ± 1.10 × 10^10^/mL, HBV: 2.66 × 10^10^/mL ± 1.32 × 10^10^/mL, HCV: 2.32 × 10^10^/mL ± 1.46 × 10^10^/mL) (Figure 2B,D). However, the concentration of MVs larger than 300 nm was significantly higher in men with HBV compared with men with HIV (3.84 × 10^8^/mL ± 2.96 × 10^8^/mL vs. 1.24 × 10^8^/mL ± 2.61 × 10^8^/mL, *p* = 0.0243) (Figure 2C). These findings indicate that, among male patients, infection type influences MV size and the abundance of larger vesicle subpopulations, particularly in HBV infection.

### 2.4. Distribution of Microvesicle Characteristics Among Female Patients by Infection Type

**Figure 3:**
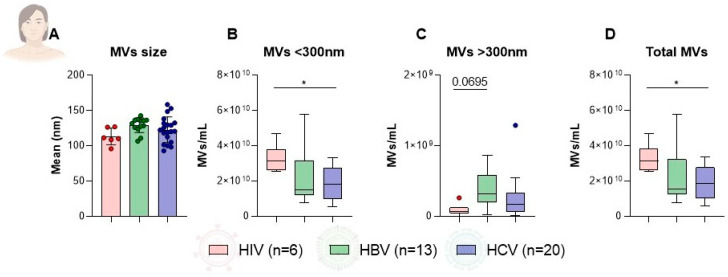
Comparison of plasma microvesicle (MV) characteristics among female patients with HIV, HBV, and HCV infections. Bar and box plots illustrate the distribution of (**A**) MV size (nm) and concentrations (MVs/mL) for particles (**B**) smaller than 300 nm, (**C**) larger than 300 nm, and (**D**) total MVs. Error bars represent standard deviation, and asterisks indicate levels of statistical significance. (* *p* < 0.05). MV, microvesicle; HIV, human immunodeficiency virus; HBV, hepatitis B virus; HCV, hepatitis C virus; nm, nanometer; mL, milliliter; *p*, probability value.

The analysis of plasma-derived MVs across HIV, HBV, and HCV infection groups revealed similar trends between males and females. The mean MV size did not significantly differ among women with HBV, HCV, and HIV (HIV: 113.4 ± 11.82 nm, HBV: 129.30 ± 10.49 nm, HCV: 123.00 ± 18.17 nm) (Figure 3A). Focusing on MV concentrations, statistically significant differences were detected between female patients with HIV and HCV for MVs smaller than 300 nm (3.27 × 10^10^/mL ± 7.87 × 10^9^/mL vs. 1.85 × 10^10^/mL ± 9.15 × 10^9^/mL, *p* = 0.0262) or for the total MV concentration (3.28 × 10^10^/mL ± 7.89 × 10^9^/mL vs. 1.88 × 10^10^/mL ± 9.32 × 10^9^/mL, *p* = 0.0305) (Figure 3B,D). Female patients with HBV exhibited a trend of higher concentration of MVs larger than 300 nm compared to women with HIV (3.83 × 10^8^/mL ± 2.89 × 10^9^/mL vs. 9.19 × 10^7^/mL ± 8.84 × 10^9^/mL, *p* = 0.0695) (Figure 3C). These results suggest that, in female patients, infection type may also affect MV size and the abundance of larger vesicle populations, particularly in HCV infection.

### 2.5. Comparison of Microvesicle Characteristics Between Male and Female Patients by Infection Type

**Figure 4:**
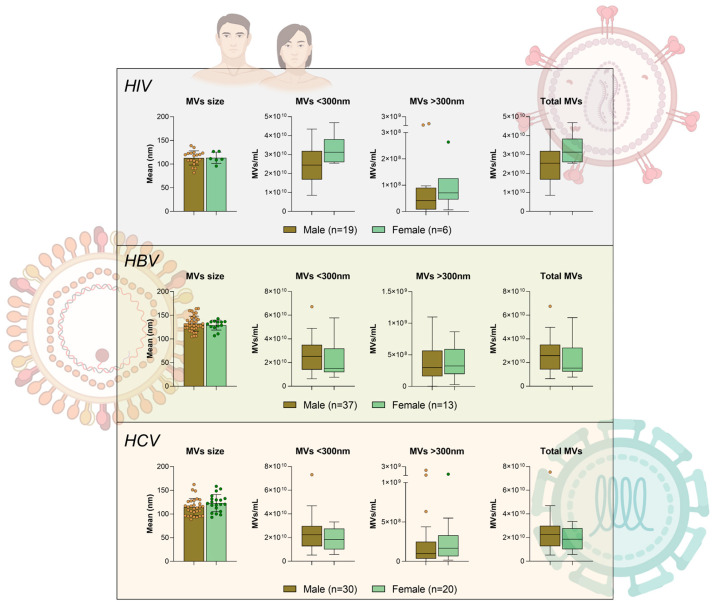
Comparison of plasma microvesicle (MV) characteristics between male and female patients with HIV, HBV, and HCV infections. Bar and box plots depict the mean MV size (nm) and concentrations (MVs/mL) for MVs < 300 nm, MVs > 300 nm, and total MVs, stratified by sex. Each infection group (HIV, HBV, HCV) is represented in separate panels, with male patients shown in brown and female patients in green. No significant differences were observed between males and females for any MV parameter across the infection types. MV, microvesicle; HIV, human immunodeficiency virus; HBV, hepatitis B virus; HCV, hepatitis C virus; nm, nanometer; mL, milliliter.

The comparison of plasma-derived MVs between male and female patients across HIV, HBV, and HCV infection groups revealed no statistically significant sex-based differences in MV size or concentration. Across all infection types, the mean MV size (nm) was similar between men (HIV:113.00 ± 14.96 nm, HBV: 132.30 ± 15.88 nm, HCV: 114.60 ± 18.19 nm) and women (HIV: 113.4 ± 11.82 nm, HBV: 129.30 ± 10.49 nm, HCV: 123.00 ± 18.17 nm), with no statistically significant differences observed. Likewise, MV concentrations for particles smaller than 300 nm, larger than 300 nm, and total MVs (MVs/mL) did not differ significantly between male and female patients within any infection group. Specifically, for MVs smaller than 300 nm, the counts were as follows: males with HIV: 2.47 × 10^10^/mL ± 1.10 × 10^10^/mL vs. females with HIV: 3.27 × 10^10^/mL ± 7.87 × 10^9^/mL; males with HBV: 2.63 × 10^10^/mL ± 1.31 × 10^10^/mL vs. females with HBV: 2.18 × 10^10^/mL ± 1.47 × 10^9^/mL; males with HCV: 2.29 × 10^10^/mL ± 1.43 × 10^10^/mL vs. females with HCV: 1.85 × 10^10^/mL ± 9.15 × 10^9^/mL. For the total MVs the counts were as follows: males with HIV: 2.49 × 10^10^/mL ± 1.10 × 10^10^/mL vs. females with HIV: 3.28 × 10^10^/mL ± 7.89 × 10^9^/mL; males with HBV: 2.66 × 10^10^/mL ± 1.32 × 10^10^/mL vs. females with HBV: 2.22 × 10^10^/mL ± 1.48 × 10^9^/mL; males with HCV: 2.32 × 10^10^/mL ± 1.46 × 10^10^/mL vs. females with HCV: 1.88 × 10^10^/mL ± 9.32 × 10^9^/mL. For MVs larger than 300 nm, the counts were as follows: males with HIV: 1.24 × 10^8^/mL ± 2.61 × 10^8^/mL vs. females with HIV: 9.19 × 10^7^/mL ± 8.84 × 10^9^/mL; males with HBV: 3.84 × 10^8^/mL ± 2.96 × 10^8^/mL vs. females with HBV: 3.83 × 10^8^/mL ± 2.39 × 10^8^/mL; males with HCV: 2.42 × 10^8^/mL ± 4.34 × 10^8^/mL vs. females with HCV: 2.45 × 10^8^/mL ± 2.95 × 10^8^/mL (Figure 4).

## 3. Discussion

MVs are known to increase or decrease in relation to stress-induced response [4,8,12], and they have also been linked to the pathogenesis of viral infections by promoting virus dissemination, inflammation, and immunomodulation [20,21]. Consistent with other studies, we found that patients with chronic viral infections exhibit different MV patterns, suggesting a role for MVs as biomarkers of viral pathophysiology and immune activation [22,23,24]. In particular, average MV size was significantly higher in patients with HBV and HCV than in those with HIV infection. This finding may reflect differences in the mechanisms of viral replication, hepatic inflammation, and cellular damage among these infections.

Interestingly, the concentration of larger MVs (>300 nm) was significantly higher in patients with HBV compared with individuals with HIV and HCV, while smaller vesicle populations and total MV counts show no major differences. Hepatocellular stress and inflammation promote the shedding of larger microvesicles alongside smaller exosomal vesicles in HBV infection. Although data remain limited, several studies report elevated levels of large plasma MVs in HBV patients, likely reflecting increased membrane budding or apoptotic vesiculation of infected hepatocytes [25,26,27,28,29].

HBV and HCV are hepatotropic and drive hepatocellular stress, inflammation, and membrane damage, promoting hepatocyte vesicle shedding across a broad EV size range, including larger microvesicles (~100–1000 nm) [30]. By contrast, HIV primarily targets immune and endothelial cells, where EV release often reflects regulated activation rather than overt cytopathic injury; e.g., gp120 or Tat can trigger endothelial microparticle release [31]. These differences in target cells, membrane perturbation, and EV biogenesis likely underlie the observed MV size discrepancies, consistent with reports that liver-derived EVs tend to be larger and more heterogeneous under inflammatory stress [32]. In the context of stress caused by HBV, microvesicles are found to be increased [33,34].

HBV-infected hepatocytes release heterogeneous EVs—exosomes (ESCRT-dependent) and microvesicles (plasma-membrane budding)—that carry viral (HBV DNA/RNA, HBsAg) and host cargos (CD81, Alix), supporting viral spread and immune modulation [28,29]. HBV EVs can facilitate immune evasion and the advancement of illness by functionally upregulating PD-L1 on monocytes and inhibiting NK cytotoxicity [26]. Although CD81’s function in HBV entrance is yet unknown, CD81^+^ EVs are widespread and can affect immune-cell activity [25,28,30,35,36]. EV cargos like miR-122. miR-21. and Hepatitis B Virus X Protein (HBx) changes the expression of host genes; EV-encapsulated HBV DNA helps identify occult HBV, and EV miR-21/miR-122 is associated with the risk of fibrosis/HCC [27,37]. Similar EV-mediated immunomodulatory and dissemination functions have been documented in HCV and HIV [38], and HBV exosomes can alter cytokine signaling (e.g., HBV-miR-3 driving macrophage IL-6) [39].

The molecular properties of HBV-associated extracellular vesicles make them potential therapeutic targets. Strategies that inhibit EV release or block EV–cell interactions are being considered to reduce viral spread and immune suppression. Furthermore, engineered exosomes carrying therapeutic cargos (e.g., siRNAs or genome editing components) are proposed as a route to selectively target HBV-infected cells [29].

Plasma EVs—including the MV-sized population—are altered in concentration and cargo composition in patients with HCV infection, reflecting disease activity and contributing to pathogenesis. For instance, a 2023 study showed that plasma EVs from HCV patients display a distinct pattern and have endothelial-damaging effects, suggesting functional roles beyond mere biomarkers [40]. In broader reviews of EV–virus interactions, HCV is known to hijack EV pathways: EVs (mainly exosomes but overlapping with MV size ranges) from HCV-infected hepatocytes carry viral RNA, proteins (e.g., E2, core), and miR-122 in complex with Ago2 and HSP90. and can transmit HCV to naïve hepatocytes otherwise shielded from neutralizing antibodies [41]. Moreover, blocking EV release in HCV cell models reduces viral replication, implying that EV (and by extension MV) release supports viral persistence and immune evasion [42]. Increased levels of platelet microvesicles in individuals with Hepatitis C could be associated with a worse clinical outcome for the patient [18].

For HIV, we know that the concentration of microvesicles in AIDS patients, whether they have received antiretroviral therapy or not, is higher than in uninfected individuals [19], due to the contribution of MVs to the spread of HIV infection [38,43] and to virus transmission [44]. In individuals with HIV, plasma contains elevated levels of EVs, including MV-sized particles, which carry host and viral cargos that can modulate immune responses and viral pathogenesis [45]. Plasma EVs from HIV+ patients have been shown to enhance HIV infection of activated CD4^+^ T cells and to reactivate latent infection, through transfer of miRNAs (e.g., miR-139-5p) that regulate factors such as FOXO1 and PD-1/PD-L1 pathways [46,47]. Moreover, EVs from HIV-infected plasma frequently harbor viral proteins such as Nef and gp120 (Env), which may facilitate viral spread or immune modulation [47,48]. Although many studies do not strictly distinguish microvesicles from exosomes, these findings suggest that plasma EV subpopulations—including MVs—could play pathological roles in HIV persistence, immune dysregulation, and viral dissemination.

When analyzing MV characteristics by gender, no significant differences were detected between male and female patients across infection types. Recent studies (particularly in HIV and hepatitis disease) have shown that sex-based differences in circulating EV or MV size and concentration are frequently negligible or inconsistent, even though sex hormones influence immune responses [44,49,50]. A subset of compartment-specific differences, such as decreased mitochondrial DNA content in neuron-derived HIV-infected male EVs, have been noted [51]; however, overall plasma EV levels seem relatively consistent between sexes. These findings suggest that, although sex may somewhat change the makeup of vesicles, it has no discernible impact on MV production or release in chronic viral infections. Overall, our findings support MVs as infection-specific markers of underlying pathophysiology. Larger size and altered concentrations in HBV/HCV likely reflect hepatic inflammation, while the absence of clear sex effects suggests infection type—rather than sex—drives MV features. Future work should define MV molecular cargo (proteins/RNAs) and clarify how it contributes to immune evasion and diagnostic utility [52,53,54].

### Limitations and Future Directions

Some clinical data, such as antiviral treatment status, viral loads, and liver function parameters, were not uniformly available across patient groups. For this reason, we were unable to perform subgroup analyses based on these variables, which might have offered additional insight into MV alterations across specific clinical categories. Future studies incorporating detailed clinical phenotyping will be essential to determine whether MV alterations correlate with specific manifestations or stages of disease. Moreover, this study analyzed only patient samples without a healthy control group, limiting the ability to define baseline MV levels. This limits direct comparison to physiological MV levels, and the observed patterns should be interpreted in this context. Furthermore, molecular characterization of MV cargo (e.g., proteins, RNA, viral components) was not performed, preventing assessment of their functional role in viral replication and immune regulation. Subsequent analyses by proteomic and transcriptomic profiling of MVs used in this manner might identify infection-specific signatures and enhance their value as possible diagnostic or prognostic markers. Inclusion of healthy controls and MV cargo profiling is planned for future validation studies.

## 4. Materials and Methods

### 4.1. Study Design and Samples

This exploratory study included 125 plasma samples from patients with infectious diseases: 50 with chronic viral hepatitis C, 50 with chronic viral hepatitis B, and 25 with human immunodeficiency virus (HIV) infection. Samples were divided into three groups according to infection type. Recorded patient data included age, sex, and diagnosis.

### 4.2. Ethical Approval and Informed Consent

This study received approval from the Research Ethics and Conduct Committee of the University of West Attica (No. PROT: 50043—21 June 2024) and from the Scientific Council of the General Hospital of Nikaia Piraeus “Agios Panteleimon” (decision No. 18, 24 April 2024; PROT: 3331—9 May 2024). Written informed consent was obtained from all participants before sample collection, in accordance with the Declaration of Helsinki. All participants agreed to the publication of the study results.

### 4.3. Sample Collection and Processing

Whole blood from patients with HBV and HCV was collected at the General Hospital of Western Attica “Agia Varvara,” and samples from patients with HIV at the General Hospital of Nikaia–Piraeus “Agios Panteleimon.” Whole blood was collected in citrate anticoagulant tubes (Becton Dickinson, USA) and centrifuged at 3000 rpm for 10 min to separate plasma. The supernatant was transferred to new tubes and centrifuged again at 3000 rpm for 10 min to obtain platelet-free plasma. Plasma aliquots were stored in sterile cryogenic microtubes at −80 °C until analysis. All procedures followed standard biosafety precautions and waste-disposal protocols.

### 4.4. MV Analysis

Before analysis, plasma samples were thawed and diluted 1:200 in filtered phosphate-buffered saline (PBS). MV concentration and size distribution were determined using a NanoSight NS300 nanoparticle tracking analyzer (Malvern Panalytical, Malvern, Worcestershire, England, UK). A syringe pump maintained a constant sample flow during measurement. Video acquisition parameters (acquisition time, brightness, and focus) were optimized for each sample. The five 30 s videos were recorded per measurement under the following conditions: cell temperature at 25 °C and syringe speed at 100 μL/s. The videos were analyzed using the NanoSight NTA 3.4 build 3.4.4 software in script control mode, with a total of 1500 frames per sample [55]. Data were processed using the instrument’s software to obtain MV size distribution and total concentration values (particles/mL) [56]. Total microvesicles (MVs) were further classified into two subpopulations according to their diameter: small MVs (<300 nm) and large MVs (>300 nm), as measured by the NanoSight NS300 analyzer. The threshold of 300 nm reflects established EV research that arose from the detection limits from flow cytometry, which could reliably measure particles only above ~200–300 nm [1]. Based on this technique-driven classification rather than a strict biological cutoff, many studies adopted this size range to distinguish larger microvesicles from smaller EVs [23]. This threshold allows the comparison with previous studies that were based on flow cytometry approach.

### 4.5. Statistical Analysis

All statistical analyses were performed using Microsoft Excel and GraphPad Prism (version 8.4.2, GraphPad Software, San Diego, CA, USA). Data were first examined for normality, and data are presented as mean ± standard deviation (SD).

Differences in MV size and concentration among the three infection groups (HIV, HBV, HCV) were assessed by one-way analysis of variance (ANOVA) followed by Tukey’s multiple-comparison post hoc test to identify pairwise differences. When data did not meet normality or equal-variance assumptions, the Kruskal–Wallis test with Dunn’s post hoc correction was applied.

Comparisons between male and female subgroups within each infection type were performed using the unpaired two-tailed Student’s *t*-test (or Mann–Whitney U test for non-normal data). A *p* < 0.05 was considered statistically significant for all analyses.

## 5. Conclusions

In summary, chronic viral infections significantly influence MV size and distribution, with HBV associated with larger MV populations compared to HIV. These findings reinforce the potential of MVs as biomarkers reflecting underlying pathophysiological processes in viral infections, particularly when correlated with established clinical and virological markers of each infection.

## Data Availability

Data is contained within the article.

## Abbreviations

The following abbreviations are used in this manuscript:

MV(s)	Microvesicle(s)
EV(s)	Extracellular Vesicle(s)
HIV	Human Immunodeficiency Virus
HBV	Hepatitis B Virus
HCV	Hepatitis C Virus
NTA	Nanoparticle Tracking Analysis
PBS	Phosphate-Buffered Saline
ANOVA	Analysis of Variance
SD	Standard Deviation
NK cells	Natural Killer cells
ESCRT	Endosomal Sorting Complex Required for Transport
PD-L1	Programmed Death-Ligand 1
miR	MicroRNA
miR-21/miR-122	MicroRNA-21/MicroRNA-122
HBx	Hepatitis B Virus X Protein
Ago2	Argonaute 2
HSP90	Heat Shock Protein 90
gp120	Glycoprotein 120
Nef	Negative Regulatory Factor
CD81	Cluster of Differentiation 81
PD-1	Programmed Cell Death Protein 1
IL-6	Interleukin-6
HCC	Hepatocellular Carcinoma
DNA	Deoxyribonucleic Acid
RNA	Ribonucleic Acid
siRNA	Small Interfering RNA
FOXO1	Forkhead Box O1
GCP	Good Clinical Practice
